# The assessment of population exposure to chlorination by-products: a study on the influence of the water distribution system

**DOI:** 10.1186/1476-069X-9-59

**Published:** 2010-10-07

**Authors:** Christelle Legay, Manuel J Rodriguez, Jean Baptiste Sérodes, Patrick Levallois

**Affiliations:** 1 École supérieure d'aménagement du territoire, Université Laval, Pavillon Antoine Savard, Québec City, QC., G1K 7P4, Canada; 2 Département de Génie Civil, Université Laval, 2917B Pavillon Pouliot, Québec City, QC., G1K 7P4, Canada; 3 Institut National de Santé Publique du Québec, 945 Avenue Wolfe, Québec City, QC., G1V 5B3, Canada

## Abstract

**Background:**

The relationship between chlorination by-products (CBPs) in drinking water and human health outcomes has been investigated in many epidemiological studies. In these studies, population exposure assessment to CBPs in drinking water is generally based on available CBP data (e.g., from regulatory monitoring, sampling campaigns specific to study area). Since trihalomethanes (THMs) and haloacetic acids (HAAs) are the most documented CBP classes in drinking water, they are generally used as indicators of CBP exposure.

**Methods:**

In this paper, different approaches to spatially assign available THM and HAA concentrations in drinking water for population exposure assessment purposes are investigated. Six approaches integrating different considerations for spatial variability of CBP occurrence within different distribution systems are compared. For this purpose, a robust CBP database (i.e., high number of sampling locations selected according to system characteristics) corresponding to nine distribution systems was generated.

**Results and conclusion:**

The results demonstrate the high impact of the structure of the distribution system (e.g., presence of intermediary water infrastructures such as re-chlorination stations or reservoirs) and the spatial variability of CBPs in the assigned levels for exposure assessment. Recommendations for improving the exposure assessment to CBPs in epidemiological studies using available CBP data from water utilities are also presented.

## Background

In recent decades, various epidemiological studies have been conducted to determine the relationship between chlorination by-products (CBPs) and different health outcomes (e.g., cancers and reproductive outcomes) [1-4]. Since trihalomethanes (THMs) and haloacetic acids (HAAs) are the most prevalent and documented CBP compounds in drinking water, they are generally considered as indicators of CBP exposure in epidemiological investigations.

In epidemiological studies focusing on THMs and HAAs in drinking water and human health outcomes, exposure misclassification can occur through the assessment of population exposure to these compounds and especially in the estimation of their levels in residential tap water [5-7]. In fact, several parameters varying in time and space, such as water source characteristics, operational parameters during treatment and distribution system specificities, influence THM and HAA occurrence in distribution systems [8]. This temporal and spatial variability within drinking water systems could result in inaccuracies in the estimation of their levels.

Generally speaking, it is difficult and expensive to obtain sufficient THM and HAA data representing these spatio-temporal variations in different distribution systems through direct measurements in residential tap water and based on a high sampling frequency. As a result, alternative data are used in epidemiological investigations on population exposure assessment. In fact, the majority of these studies use data available through regulatory compliance on CBPs in drinking water for which THMs and HAAs (the latter only in United States (US)) are routinely measured [9-17]. With some differences according to region or country, regulatory compliance generally requires a minimum of quarterly CBP measurements at one to four sampling locations per distribution system [18-20].

Given the potential spatial variability of THM and HAA occurrence within distribution systems, a major challenge facing epidemiological studies based on available THM and HAA concentrations in tap water is to spatially assign these data to subjects in order to be the most representative of real exposure. In the studies where data are available only at a single location in the distribution system, these data are assigned directly to the subject's residential tap water. However, in the case where more than one location in the subject's distribution system is sampled, different approaches are applied to estimate THM and HAA levels in the subject's residential tap water. The distribution system-wide average of THM and HAA data is used for exposure assessment in most epidemiological studies [9,11,13-15,21]. Other studies consider THM and HAA data from the closest sampling location within the distribution system to the subject's residence as representative of the subject's exposure [16,22]. Different methods to assign available data to subjects are also applied, such as the use of the distribution and statistical variance of THM and HAA levels in distribution systems [22,23]. The more accurate approach is based on the assignment to the subjects of data from the sampling location in the distribution system with hydraulic characteristics similar to the target location [10,12]. However, this type of information is rarely available. According to the degree of spatial variability of THMs and HAAs and the subject's residential location within distribution systems, these approaches could involve misclassifications in exposure assessment [13,22-24]. In fact, distribution system characteristics that influence THM and HAA occurrence, such as system size and the presence of intermediary water infrastructures to the water treatment plant (i.e., re-chlorination station or reservoir), are generally not considered. As a result, data used to assess the THM and HAA levels in the subject's tap water from a sampling location in the subject's distribution system are not necessarily representative of the subject's residential water quality (e.g., with different hydraulic characteristics and not being supplied by the same direct water infrastructure) [7].

The main objective of this paper is to investigate and compare different spatially-based approaches to assign available THM and HAA concentration data to subjects involved in epidemiological studies. These approaches integrate different considerations of the variability of THM and HAA occurrence within distribution systems. In addition, for each method, the impact of distribution characteristics (i.e., system size and structure) on the estimation of THM and HAA levels in the subject's tap water is studied. For the study, an important database in terms of population under study and CBP measurements was used to test six different approaches. The results from the assignment of THM and HAA data to the subjects following each approach are compared. Finally, recommendations are made to minimize exposure misclassifications associated with spatial variability of THMs and HAAs in drinking water in the assignment of their levels to the subject's residential tap water.

## Methods

### Case under study

**Figure 1:**
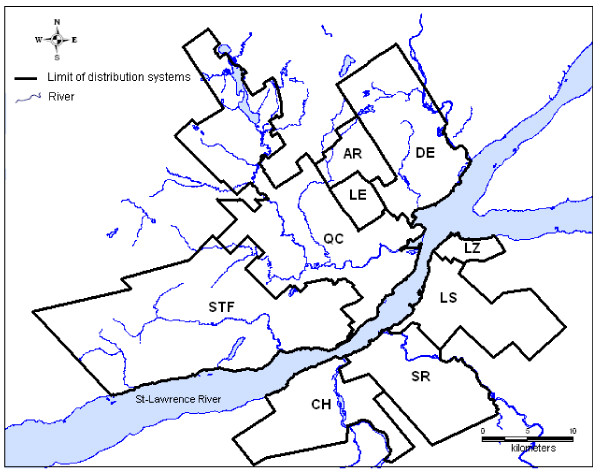
**Location of the nine distribution systems under study**.

This study was conducted with nine distribution systems of the greater area of Québec City (Province of Quebec, Canada). Five distribution systems denoted AR, DE, LE, QC and STF supply Québec City (approximately 480,000 inhabitants) and four systems denoted CH, LS, LZ and SR supply the city of Lévis (approximately 120,000 inhabitants). These systems are illustrated in Figure 1 and their main characteristics are presented in Table 1. Each distribution system is supplied by surface water and uses free chlorine for primary or secondary disinfection (that generally follows physico-chemical water treatment such as coagulation, sedimentation and filtration). However, characteristics of the supply system such as the type of water source (e.g., river and lake), water treatment processes, population served, system size and hydraulic conditions vary from one system to another. As shown in Table 1, for a specific distribution system, the population might be served by a different water infrastructure, either directly by the water treatment plant or through a re-chlorination station or reservoir. Each area served by a different water infrastructure within the same distribution system is defined as a sub-system. In the case where a distribution system is directly supplied only by the treatment plant, the presence of a single sub-system is assumed. The nine distribution systems under study differ also in the number and type of sub-systems (Table 1).

**Table 1:** Characteristics of the nine distribution systems under study

Distribution system	Water source	**Flow rate (m**^**3**^**/day)**	Number of sub-systems	Particular characteristics	Number of sampling sites	**Number of subjects**^**a**^
AR	Des Roches Lake.	± 9,000	3	Re-chlorination stations or reservoirs.	3	76 (194)

DE	Montmorency River.	± 33,000	5	Re-chlorination stations or reservoirs.	5	188 (493)

LE	Sept Ponts River.	± 19,000	1	None.	2	91 (249)

QC	St. Charles Lake.	± 145,000	4	Re-chlorination stations or reservoirs.	10	549 (1,457)

STF	St. Lawrence River.	± 69,000	5	Re-chlorination stations or reservoirs.	6	189 (498)

CH	Chaudiere River.	± 13,000	6	Re-chlorination stations or reservoirs.	8	117 (309)

LS	St. Lawrence River.	± 24,000	2	Re-chlorination stations or reservoirs.	5	120 (316)

LZ	St. Lawrence River.	± 11,000	2	Re-chlorination stations or reservoirs	3	44 (112)

SR	St. Lawrence River.	± 10,000	2	Re-chlorination stations or reservoirs.	4	96 (251)

^a ^The number of pregnancy trimesters studied is shown into parenthesis

The region under study is subject to important climatic variations during the year, with average air ambient temperature ranging from -15°C to + 24°C and different lengths of seasons (i.e., long winters and relatively short summers). These temporal fluctuations involve important temporal variations in the quality of raw and treated water.

### Description of data

#### Population under study

The population under study involved subjects from a case-control epidemiological investigation on intrauterine growth retardation (IUGR) carried out in the greater area of Québec City during 2006-2008 (Levallois et al. 2009; personal communication). The subjects were women who each delivered a single live infant for whom exposure to CBPs in drinking water during each pregnancy quarter was studied. This paper is restricted to women living in a single residence located in one of the nine systems studied during their entire pregnancy period. Moreover, only pregnancy trimesters comprised between April 2006 and April 2008 were included in the analysis. By means of a phone questionnaire carried out approximately one to two months after the women gave birth, information on their pregnancy residence address (i.e., house number, street, municipality, and postal code) was obtained. The geographical location of each subject's residence in the appropriate system and sub-system under study was determined using a geographical information system (GIS) supported by MapBasic Version 8.0 that integrates residential postal codes (with Platinum Postal Suite™ 2008.3).

This paper focuses specifically on the spatial assignment of CBP data in residential tap water for each subject. The spatial variability of THMs and HAAs within distribution systems can be influenced by temporal variations (i.e., seasons, months) of water quality [25]. In order to minimize the temporal impact of the occurrence of THMs and HAAs on the subject's exposure assessment obtained from the different spatial approaches, multiple exposure periods were investigated for each subject. These periods corresponded to different trimesters of their pregnancy (varying from two to three according to the subjects) and thus to different seasonal periods of the year. A total of 3,879 pregnancy trimesters for 1,470 subjects were considered in this investigation (Table 1).

#### CBP data collection

The estimation of THM and HAA levels in a subject's tap water during each exposure period under study was based on data from sampling campaigns tailor-made for the epidemiological study on IUGR. Between April 2006 and April 2008, monthly sampling campaigns for THM and HAA measurements were carried out at 46 sampling sites spatially distributed in the nine distribution systems. The number and location of the sampling sites were specifically selected according to the size of the population served and system structure (e.g., according to system size and number of sub-systems). For the nine distribution systems, at least one sampling site was located in each sub-system. The distribution of the number of sampling sites among the nine systems is also presented in Table 1.

For THMs, the four following compounds were analyzed: chloroform, bromodichloromethane (BDCM), dibromochloromethane (DBCM) and bromoform. The sum of these four compounds represents the total THMs (TTHMs). The nine individual HAAs were also analyzed: monochloroacetic (MCAA), dichloroacetic (DCAA), trichloroacetic (TCAA), monobromoacetic (MBAA) and dibromoacetic (DBAA), tribromoacetic (TBAA), bromochloroacetic (BCAA), dibromochloroacetic (DBCAA), bromodichloroacetic (BDCAA) acids. HAA9 refers to the sum of these nine compounds. The sampling and analytical procedures applied to characterize the THM and HAA levels are described in a previous study [26]. Measurements below their detection limit for each compound under study were considered equal to zero [22,23]. In the rest of paper, CBPs refer to TTHMs and HAA9.

### Assessment of CBP levels in the subject's tap water

Six different methods were applied and compared to estimate CBP levels in the subject's drinking water. In the six methods denoted A to F, the spatial variability of THMs and HAAs in drinking water was integrated differently. These methods differ in various ways: geographical scale considered (i.e., the entire distribution system or the sub-system), number of sampling sites considered and location of sampling sites associated with the subject's residence. Moreover, the metric applied to assign the CBP data from sampling site(s) selected to the subjects (i.e., direct assignment of CBP data, average or use of weighting factor) also differs according to the methods. The metric generally depends on the number of sampling sites selected. Since the geographical scale significantly influences the other factors (i.e., the number and the location of sampling sites), the different methods under study are presented according to the geographical scale under consideration.

#### Methods applied at the sub-system scale

In four of the six CBP level assessment methods under study, only the sampling site(s) supplied by the same direct water infrastructure of the subject's residence (i.e., located in the subject's sub-system) were considered: methods A, B, C and D.

Method A: The two sampling sites closest to the subject's residence were used. In order to integrate the potential spatial variations of CBP occurrence within sub-systems, a weighting factor was applied at these two sampling sites. The weighting factors were estimated on the basis of the distance between the subject's residence and the two specified sampling sites. As a result, for each of the sampling sites considered, the weighting factors (WF) were calculated as following:

(1) $$WF_{P1}=1-(\frac{d_{P1}}{d_{P1}+d_{P2}})$$

(2) $$WF_{P2}=1-(\frac{d_{P2}}{d_{P1}+d_{P2}})$$

where P_1 _and P_2 _are the first and the second specified sampling sites respectively, that represent CBP exposure of a subject, and d denotes the distance between the specified sampling site and the subject's residence (without unit: distances were standardized from coordinates).

Method B: The average of CBP data from the two closest sites to the subject's residence was carried out (i.e., method A without weighting factor) and assigned to the subjects.

Method C: CBP data from the closest sampling to the subject's residence were directly assigned to the subjects.

Method D: The average of CBP data from all sampling sites located in the sub-system was carried out and assigned to the subjects.

In various sub-systems, a single location was sampled. As a result, irrespective of the method applied at the sub-system scale, the CBP data from this site were considered to represent exposure for subjects located in the sub-system.

#### Methods applied at the distribution system-scale

The two remaining methods (methods E and F) considered sampling sites located in the same distribution system of the subject's residence.

Method E: CBP data from the closest sampling site to the subject's residence were assigned directly to the subjects (i.e., without considering the presence of an intermediary water infrastructure). This approach has also been applied to assign CBP data to the subjects in several epidemiological studies [16,22].

Method F: The average of CBP data from all sampling sites located in the distribution system was calculated and assigned to the subjects. This method represents the usual spatial approach applied to assign available CBP data to the subjects in epidemiological studies [9,11,13-15].

In these methods applied at the two geographical scales, the road distance from the subject's residence to a specific sampling site was used as an indicator of proximity (not used in the methods D and F). However, the systematic correlation between the distance to a sampling site and the CBP levels was not demonstrated [22]. Since this type of proximity indicator was available (contrary to the precise water residence time in the pipes), it was used in the CBP level assessment methods presented herein.

For each method, the estimation of CBP levels in the subject's tap water through the assignment of available CBP data in this study was performed using SAS Version 9.2 [27].

### Data analysis

In order to compare CBP levels between years, sub-systems and systems, several statistical tests were carried out. Since TTHM and HAA9 levels were not always distributed in accordance with the Gaussian Law, non-parametric tests were used. As a result, the Kolmogorov-Smirnov Z test was applied when two independent samples were compared and the Kruskal-Wallis H test when more than two independent samples were compared. Each of these tests was carried out using the statistical significance level ρ < 0.05 (SPSS Version 13.0).

Usually, in epidemiological studies, the relationship between adverse health outcomes and CBPs in drinking water is evaluated using categorical exposure variables. The categories of CBP exposure are generally based on CBP levels measured in the area under study and more precisely on CBP level percentiles [14,17,21]. As a result, for each of the six methods under study, the subjects were classified in TTHM and HAA9 exposure categories according to the estimation of CBP levels in their residence drinking water obtained from the specific method. TTHM and HAA9 exposure categories were based, as thresholds, on quartiles of their levels measured in the drinking water distribution systems. A first analysis including all the distribution systems was carried out (i.e., the cut-points based on the CBP levels measured in all the distribution systems). In order to evaluate the impact of the methods according to the characteristics of the distribution system, analyses for each individual system were also carried out with exposure categories specific to each system.

Kappa statistics were estimated to compare the classification of the subjects in TTHM and HAA9 exposure categories obtained from the six methods. The Kappa coefficient represents the agreement between two methods [28,29]. Given the ordered nature of outcomes (i.e., exposure categories), weighted kappa coefficients (κ) were estimated with a linear weighting scheme [30]. For each compared pair-method (e.g., methods A and B, methods A and C, methods B and C), κ value was calculated using the statistical level ρ < 0.05 (STATA Version 9.0). The agreement between two methods was considered as "poor" for κ ≤ 0.20; "Fair" for 0.21 ≤ κ ≤ 0.40; "moderate" for 0.41 ≤ κ ≤ 0.60; "substantial" for 0.61 ≤ κ ≤ 0.80; and "almost perfect" with 0.81 ≤ κ ≤ 1.00 [31].

## Results and discussion

### CBP occurrence in the area under study

Table 2 presents the TTHM and HAA9 levels measured during the 2006-2008 period in the nine distribution systems under study. As shown in this table, average CBP levels during the entire study period differed from one distribution system to another. In fact, the highest average TTHM levels were found for the LE, AR and DE systems. The regulatory standard for TTHMs is 80 μg/L in the Province of Quebec and in US [18,20]. Average TTHM levels were not consistent with regulatory compliance for the DE system in 2006-2007 and in 2007-2008 and for the AR and LE systems in 2007-2008. Lower TTHM concentrations were observed for the other systems and principally for the QC and STF systems. A similar pattern was observed for HAA9 with levels below those measured for TTHM, except for the AR, DE and QC systems (Table 2). Levels above the US standard for HAA5 (which is the sum of MCAA, DCAA, TCAA, MBAA and DBAA) of 60 μg/L [20] were found (data not shown) for the same systems and the same periods as the TTHMs. The temporal variations of TTHM and HAA9 occurrence in drinking water were also investigated. To achieve this, average CBP levels were compared for each distribution system for 2006-2007 and 2007-2008. In the case of HAA9, statistically significant different levels were observed between the two years for the DE, LZ and SR systems. Only two systems (LE and SR) presented a statistically significant difference between the two average annual TTHM levels.

**Table 2:** CBP levels measured in each distribution system during 2006-2008

Distribution system	TTHM (μg/L)					HAA9 (μg/L)				
	**25**^**th **^**Perc**.^**a**^	**50**^**th **^**Perc**.	**75**^**th **^**Perc**.	**Mean**	**SD**^**b**^	**25**^**th **^**Perc**.	**50**^**th **^**Perc**.	**75**^**th **^**Perc**.	**Mean**	**SD**

AR	31.1	67.4	117.5	76.6	50.0	36.2	60.4	100.9	74.9	50.0

DE	61.8	99.1	162.7	111.3	61.2	73.5	113.5	145.1	115.2	52.9

LE	43.4	61.8	96.7	75.2	43.0	43.5	56.4	77.4	66.5	33.7

QC	15.1	22.5	34.0	26.0	14.5	16.7	23.0	31.6	25.3	11.3

STF	26.8	35.4	47.3	38.1	15.0	21.6	28.6	36.1	29.2	10.5

CH	30.0	45.5	79.1	57.0	33.3	34.9	45.4	58.0	48.4	19.4

LS	29.9	40.3	53.4	42.1	16.7	24.7	31.9	38.9	32.3	9.9

LZ	32.1	39.4	60.8	46.8	20.2	30.4	34.9	41.4	36.0	9.1

SR	39.2	57.6	75.4	59.4	23.7	31.8	43.4	55.6	45.6	17.4

^a^Perc.: percentile

^b^SD: standard deviation

**Figure 2:**
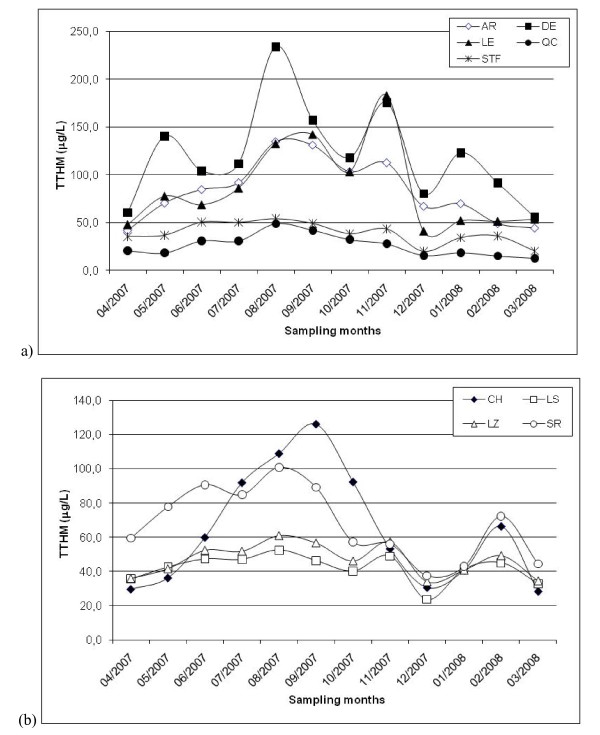
**Monthly TTHM levels measured in distribution systems during 2007-2008 (a) Québec City; (b) City of Lévis**.

**Figure 3:**
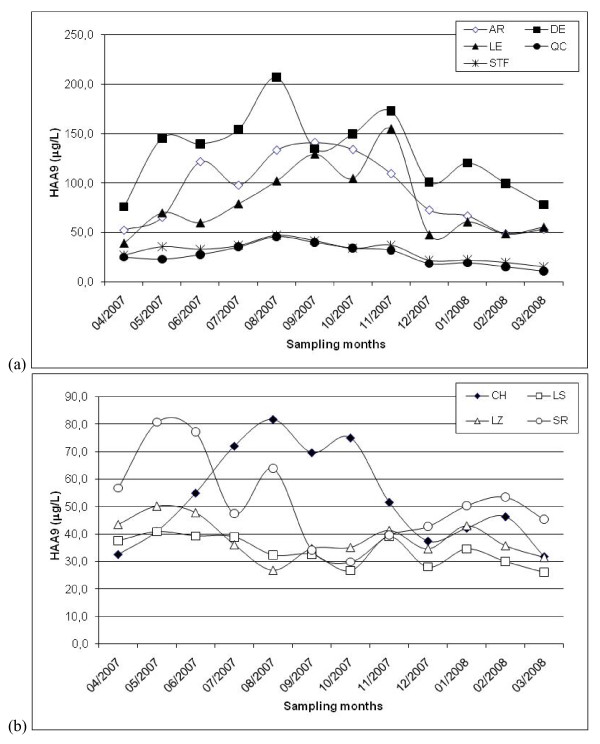
**Monthly HAA9 levels measured in distribution systems during 2007-2008 (a) Québec City; (b) City of Lévis**.

As shown in Table 2, the distribution of TTHM and HAA9 levels (i.e., represented by percentile and standard deviation values) found for each distribution system during the entire period is widespread. Moreover, this phenomenon is more obvious in the AR, DE, LE, SR and CH systems. One explanation for these results was the presence of a substantial temporal variability of water quality during the year in the Québec region. For example, Figures 2a-b illustrate the statistically significant seasonal variations of TTHM levels in 2007-2008. A comparable temporal pattern of TTHM variability was observed for the nine distribution systems for which higher TTHM levels were measured during summer and fall. In the case of HAA9 occurrence, statistically significant temporal variations were also obtained (Figures 3a-b). However, the temporal pattern differed between the distribution systems.

**Figure 4:**
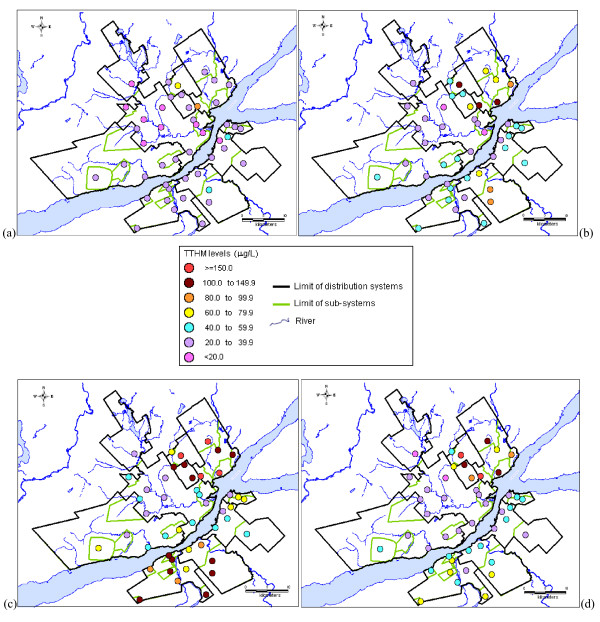
**Quarterly average of TTHM levels measured in the nine distribution systems during 2007 (a) 1^st ^quarter; (b) 2^nd ^quarter; (c) 3^rd ^quarter; (d) 4^th ^quarter**.

**Figure 5:**
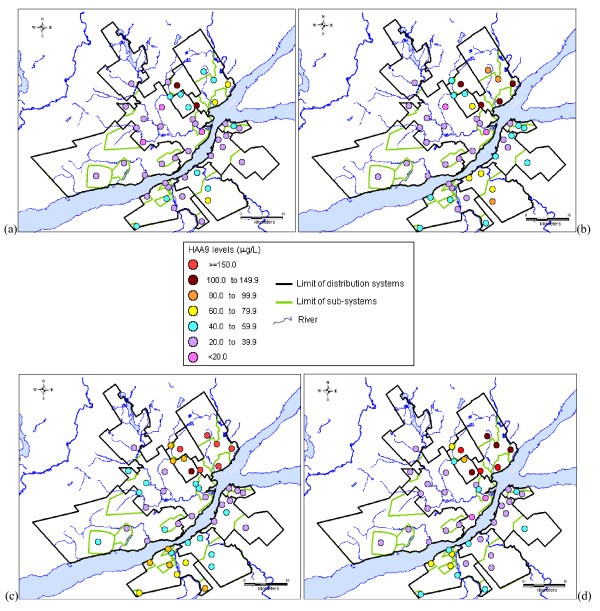
**Quarterly average of HAA9 levels measured in the nine distribution systems during 2007 (a) 1^st ^quarter; (b) 2^nd ^quarter; (c) 3^rd ^quarter; (d) 4^th ^quarter**.

Another explanation for the widespread distribution of CBP levels measured for the distribution systems is the presence of the spatial variability of CBP occurrence within these systems. Figures 4 and 5 present the quarterly average of TTHM and HAA9 levels measured in all sampling sites during 2007, respectively. These figures confirm the statistically significant differences of average CBP levels between the sub-systems of each distribution system (except for the LS system). Figures 4a-d also illustrate the variability within sub-systems of TTHM levels for most systems. The spatial variability between and within sub-systems was also observed for HAA9, but with different patterns (Figures 5a-d). The spatial variability of TTHM and HAA9 levels confirms the influence of the distribution system structure on CBP occurrence, such as the number and the nature of sub-systems (i.e., the type of water supply infrastructure) and the sub-system location within the systems.

In addition, Figures 4 and 5 show that the variability pattern of CBP occurrence within distribution system differed according to the quarter under study. For example, the spatial variability of TTHM levels within distribution systems was relatively low during the 1^st ^quarter, which corresponds to the winter (Figure 4a), as compared to the 3^rd ^quarter (Figure 4c). Figures 5a-d also demonstrate the difference of spatial variations of HAA9 occurrence within distribution systems between the quarters of the year. Combined with the presence of monthly variations of TTHM and HAA9 occurrence (Figures 2 and 3), these results justify the study of more than one pregnancy trimester for each subject.

### Comparison of methods used to spatially assign CBP data to the subjects

The previous section demonstrated the high spatial variability of TTHM and HAA9 occurrence within distribution systems, with the degree dependant mainly on structural characteristics and treated water quality. The non-consideration of CBP variations within distribution systems in the assessment of CBP levels in the subject's tap water based on available data in epidemiological studies could result in exposure assessment misclassifications.

For each method, the subjects were classified in CBP exposure categories according to the estimated CBP levels in their residential tap water. The classification of the subject's exposure to TTHM and HAA9 specific to each method was first carried out on a regional basis, considering the entire area under study (i.e., the nine distribution systems). Since the range of measured CBP levels and the degree of spatial variability of their occurrence differed from one distribution system to the other, the classification of the subject's exposure by the six methods also was carried out specifically for each individual distribution system.

#### Comparison of methods considering the entire area under study

As described in Section 2.4, the four following TTHM exposure categories were created according to TTHM levels measured in the entire area: ≤ 26.8 μg/L (1^st^); 26.9 to 40.9 μg/L (2^nd^); 41.0 to 67.6 μg/L (3^rd^); ≥67.7 μg/L (4^th^). In the same way, four HAA9 exposure categories were obtained: ≤ 25.7 μg/L (1^st^); 25.8 to36.5 μg/L (2^nd^); 36.6 to 53.5 μg/L (3^rd^); ≥53.6 μg/L (4^th^). κ values representing the agreement between the classification of the subjects in the TTHM and HAA9 exposure categories obtained from the six assignment methods are presented in Table 3.

**Table 3:** κ values for the subject's classification in the CBP exposure categories obtained from the six assignment methods considering the entire area under study

CBP studied	Comparison of methods applied at the same space scale							Comparison of methods applied at a different space scale							
	**Sub-system**						**System**								

	**A-B**	**A-C**	**A-D**	**B-C**	**B-D**	**C-D**	**E-F**	**A-E**	**B-E**	**C-E**	**D-E**	**A-F**	**B-F**	**C-F**	**D-F**

TTHM	0.98	0.94	0.95	0.92	0.96	0.92	0.72	0.86	0.84	0.90	0.82	0.74	0.75	0.71	0.77
HAA9	0.97	0.95	0.93	0.92	0.94	0.90	0.70	0.86	0.83	0.90	0.81	0.73	0.73	0.70	0.72

As shown in Table 3, substantial levels of agreement between the methods applied at different space scales were observed for TTHMs and HAA9. These results are explained mainly by the high spatial variability of CBP occurrence in distribution systems. Consequently, the type of space scale considered in the CBP data assignment method has an impact on the subject's exposure classification.

Table 3 demonstrates that the spatial variability of CBPs in drinking water also involves differences in the exposure classification between the methods based on one or two sampling sites (whatever the scale considered) and the method based on system-wide sampling (method F).

The results from the methods applied at the sub-system scale were relatively similar with κ values above 0.92 for TTHMs and 0.90 for HAA9 (Table 3). These results show that the spatial variability of CBP occurrence within sub-systems has a small impact on the exposure classification when the entire area under study is considered. The small number of sampling sites for most sub-systems could also explain these results (rarely > 2). Considering the entire area under study, the selection of one method among the four methods at the sub-system scale had little impact on the classification of the subjects in CBP exposure categories.

κ values from the comparison between the two methods based on the closest sampling site, but at a different scale (i.e., considering only the sub-system for method C and the entire system for method E), were 0.90 for both TTHMs and HAA9. As a result, when the entire region under study was considered, the closest sampling site in the system of the subject's residence was generally representative of the water quality of the direct water supply infrastructure (e.g., re-chlorination station or the water treatment plant).

In this section, the six exposure assessment methods were compared on the basis of the entire area under study (the nine systems considered together). Thus, the spatial variability of CBP occurrence within specific systems was not taken into account. Since the TTHM and exposure categories were based on levels measured in the entire area, the differences in the range of measured CBP levels between systems could bring about a bias on the observed impact of the assignment method.

#### Comparison of methods for each individual distribution system

In order to classify the subject's exposure with each method, four exposure categories were created for each distribution system according to quartiles of CBP levels measured in the specific systems (Table 2). Tables 4 and 5 present for each distribution system, the κ values corresponding to the comparison of the classification of the subject's exposure to TTHM and HAA9 obtained from the six methods.

**Table 4:** κ values for the subject's classification in the TTHM exposure categories obtained from the six assignment methods for each distribution system

System name	Comparison of methods applied at the same space scale							Comparison of methods applied at a different space scale							
	**Sub-system**						**System**								

	**A-B**	**A-C**	**A-D**	**B-C**	**B-D**	**C-D**	**E-F**	**A-E**	**B-E**	**C-E**	**D-E**	**A-F**	**B-F**	**C-F**	**D-F**

AR	1.00	1.00	1.00	1.00	1.00	1.00	0.07	0.63	0.63	0.63	0.63	0.02	0.09	0.09	0.09

CH	0.98	0.96	0.98	0.94	1.00	0.94	0.66	0.86	0.84	0.90	0.84	0.64	0.64	0.65	0.64

DE	1.00	1.00	1.00	1.00	1.00	1.00	0.56	0.82	0.82	0.82	0.82	0.52	0.52	0.52	0.52

LZ	0.99	0.97	0.99	0.96	1.00	0.96	0.22	0.71	0.70	0.74	0.70	0.12	0.13	0.14	0.13

STF	1.00	1.00	1.00	0.99	1.00	0.99	0.52	0.58	0.58	0.59	0.58	0.39	0.39	0.39	0.39

LE	0.93	0.85	0.93	0.78	1.00	0.78	0.78	0.85	0.78	1.00	0.78	0.93	1.00	0.78	1.00

SR	0.94	0.92	0.94	0.85	1.00	0.85	0.61	0.90	0.84	0.98	0.84	0.58	0.58	0.60	0.58

LS	0.79	0.70	0.43	0.50	0.47	0.37	0.27	0.70	0.50	1.00	0.37	0.34	0.38	0.27	0.81

QC	0.92	0.87	0.77	0.79	0.80	0.72	0.51	0.82	0.77	0.89	0.67	0.57	0.60	0.52	0.71

**Table 5:** κ values for the subject's classification in the HAA9 exposure categories obtained from the six assignment methods for each distribution system

System name	Comparison of methods applied at the same space scale							Comparison of methods applied at a different space scale							
	**Sub-system**						**System**								

	**A-B**	**A-C**	**A-D**	**B-C**	**B-D**	**C-D**	**E-F**	**A-E**	**B-E**	**C-E**	**D-E**	**A-F**	**B-F**	**C-F**	**D-F**

AR	1.00	1.00	1.00	1.00	1.00	1.00	0.14	0.61	0.61	0.61	0.61	0.13	0.13	0.13	0.13

CH	0.98	0.95	0.98	0.92	1.00	0.92	0.54	0.80	0.78	0.85	0.78	0.54	0.55	0.55	0.55

DE	1.00	1.00	1.00	1.00	1.00	1.00	0.45	0.83	0.83	0.83	0.83	0.45	0.45	0.45	0.45

LZ	0.98	0.95	0.98	0.93	1.00	0.93	0.35	0.68	0.66	0.72	0.66	0.37	0.36	0.35	0.36

STF	0.99	0.97	0.99	0.96	1.00	0.96	0.35	0.46	0.45	0.49	0.45	0.31	0.32	0.30	0.32

LE	0.91	0.84	0.91	0.75	1.00	0.75	0.75	0.84	0.75	1.00	0.75	0.91	1.00	0.75	1.00

SR	0.93	0.90	0.93	0.83	1.00	0.83	0.79	0.88	0.82	0.98	0.82	0.84	0.86	0.79	0.86

LS	0.86	0.84	0.73	0.70	0.80	0.60	0.31	0.84	0.70	1.00	0.60	0.37	0.39	0.31	0.44

QC	0.91	0.83	0.75	0.74	0.76	0.67	0.32	0.78	0.70	0.85	0.62	0.43	0.45	0.31	0.48

For the AR, CH, DE, LZ and STF systems, high κ values were observed for the classification of subject's exposure to TTHM obtained from methods applied at the sub-system scale (Tables 4 and 5). For the DE and AR systems, κ values were due to the presence of a single sampling location per sub-system. In the case of the CH, LZ and STF systems, the high reliability between the methods was associated with the low variability of TTHM occurrence within sub-systems which is generally due to their small size. However, the relative structural complexity of these systems involves important TTHM level variations within the entire system (i.e., between the sub-systems). As a result, important differences between the system-wide average method (method F) and the five other methods were observed (κ values < 0.10). As shown in Table 5, a similar pattern was observed for HAA9 with a different intensity according to the system.

Since the LE system consists of a single system, methods C and E are similar (same thing for methods D and F). In order to facilitate the result analysis, only methods A to D were compared for this system. The κ values obtained from the comparison between these four methods show a slight impact of the method on the classification of the subject's exposure to TTHMs and HAA9. These results are probably due to the structural simplicity of this system (one sub-system) and to the low variations of CBPs within this system.

The SR and LS systems include two different sub-systems (one directly supplied by the water treatment plant and the other supplied by a re-chlorination station) which involve spatial variations of TTHMs and HAA9 in the entire system. As shown in Tables 4 and 5, these variations have an impact on the classification of the subject's exposure according to the method applied. For example, exposure results with the method based on a single sampling site at the system scale (method E) differed from the results with the method based on the system wide-average (method F). This phenomenon was particularly pronounced for the LS system (Table 4). The impact of spatial variations of TTHM levels within sub-systems on the subject's exposure assessment was also observed for these two systems. In fact, κ values below 0.50 for the LS system and of 0.85 for the SR system were obtained from the comparison at the sub-system scale between the method using a single sampling site (method C) and the methods using two or more sampling sites without distance factor (methods B and D). A comparable pattern was observed for HAA9.

As shown in Tables 4 and 5, the space scale has an impact on the classification of the subject's exposure in the QC system with relatively low levels of agreement between the methods at the sub-system scale and the methods at the system scale. The differences obtained between the four methods at the sub-system scale demonstrate the influence of the proximity on the subject's classification in the QC system. However, as shown in Table 2, CBP levels measured in the QC system were relatively low. As a result, differences observed between the methods could be due primarily to the low range of CBP levels and may not reflect the real impact of the method applied to the subject's exposure assessment.

For each of the nine distribution systems under study, important differences were observed between the subject's exposure classification in the two methods based on the closest sampling site, but at a different space scale (methods C and E). As a result, the sampling site that considers the entire system might not be representative of the water quality from the direct subject's supply infrastructure (e.g., re-chlorination station) and could induce misclassification for CBP exposure estimation. As shown in Tables 4 and 5, the level of agreement between methods C and E varies according to the spatial variability of TTHM and HAA9 levels in the distribution system.

## Conclusions

This paper shows that the spatial approach applied to assign TTHM and HAA9 data to subjects has an important impact on exposure assessment results. The non-consideration of this variability in the assessment of CBP levels in the subject's drinking water could result in exposure misclassification.

According to the results obtained in this study, some recommendations can be made to improve the assessment of the subject's exposure based on available TTHM and HAA9 data in epidemiological studies and thus to minimize exposure misclassification (Table 6). These recommendations are adapted according to the spatial variability of CBP occurrence within systems (which could be estimated through available data from other studies or from regulatory surveys) and to the type of data available. As shown in this study, the spatial variability of CBPs differs according to systems (influence of the treated water quality and system structure). As a result, the approach used to assign available CBP data to subjects should be specific to each system. Moreover, specific considerations should be afforded to each CBP class under study.

**Table 6:** Recommendations to spatially assign the available CBP data to the subjects in epidemiological studies

Degree of spatial variability of CBP levels in the system	Number of CBP data available for the distribution system under study		
	**1 site/sub-system**	**> 1 site/sub-system**	**> 1 site/distribution system (but not sampling site in each sub-system)**

High	Use of the sampling site in sub-system	The closest sampling site from the subject's residence	Determination of comparable sub-systems. Use of the sampling site located in sub-system with the most similar characteristics.

Low	Use of the sampling site in sub-system	Average of all sampling sites or average of the two closest sites from the subject's residence	Similar to above or average of all sampling sites

In this study, the number of sampling sites per sub-system differed according to the system. This could affect the comparison of the classification of the subject's exposure to CBP (obtained from each method) between systems. Moreover, only spatial aspects were considered in the assignment of CBP data to subjects. However, as shown in Section 3.1, the considerable temporal variability of THMs and HAAs was also demonstrated. This temporal aspect should be considered in the assessment of the CBP levels in the subject's drinking water, particularly in the case where short-term exposure periods are studied (e.g., studies on adverse reproductive outcomes).

THMs and HAAs are not always correlated with other CBP compounds [32]. As a result, the relevance of their use as markers of CBP exposure could be questioned. In future studies, the assessment of population exposure to CBPs in drinking water assessment should include the measurement of other CBPs (e.g., haloacetonitriles, haloketones) [1]. Since the mechanisms of toxic action differ from one compound of the same CBP class to another (e.g., individual THMs or HAAs), the effect of individual CBPs should be investigated [33-35]. Future research should also focus on the effects of the mixtures of CBPs in drinking water on human health [32,33].

## List of abbreviations

BDCM: Bromodichloromethane; BCAA: Bromochloroacetic acid; BDCAA: Bromodichloroacetic acid; CBP: Chlorination by-products; DBCAA: Dibromochloroacetic; DBCM: Dibromochloromethane; DBAA: Dibromoacetic acid; DCAA: Dichloroacetic acid; GIS: Geographical information system; HAA: Haloacetic acids; HAA 9: Sum of the nine haloacetic acids; IUGR: Intrauterine growth retardation; MCAA: Monochloroacetic acid; MBAA: Monobromoacetic acid; PERC.: Percentile; SAS: Statistical analysis system; SD: Standard deviation; SPSS: Statistical package for the social sciences; TBAA: Tribromoacetic acid; TCAA: Trichloroacetic acid; THM: Trihalomethanes; TTHM: Total trihalomethanes; US: United states; WF: Weighting factor.

## Competing interests

The authors declare that they have no competing interests.

## Authors' contributions

CL conceived and designed the study, collected the data, performed the statistical analysis, interpreted the data and drafted the manuscript; MJR contributed to conception of the study and its design, supervised the study and helped to draft the manuscript, JBS and PL contributed to conception of study and helped to interpret the data. All authors read and revised the final manuscript.
